# Does endometrial receptivity array improve reproductive outcomes in euploid embryo transfer cycles? a systematic review

**DOI:** 10.3389/fendo.2023.1251699

**Published:** 2023-10-23

**Authors:** Youwen Mei, Yacong Wang, Xue Ke, Xuefei Liang, Yonghong Lin, Fang Wang

**Affiliations:** Department of Reproduction and Infertility, Chengdu Women’s and Children’s Central Hospital, School of Medicine, University of Electronic Science and Technology of China, Chengdu, China

**Keywords:** endometrial receptivity array, euploid embryo transfer, *in vitro* fertilization, recurrent implantation failure, reproductive outcomes

## Abstract

Besides chromosomal normality, endometrial receptivity is an important factor in determining successful pregnancies. Endometrial receptivity array (ERA), a promising endometrial receptivity test, was speculated to improve the reproductive outcomes. However, its effectiveness is controversial in clinical practice. Therefore, we conducted this review to investigate its role in *in vitro* fertilization (IVF) treatment. To eliminate the interference of embryo quality, we only analyzed studies that originally reported the reproductive outcomes of patients who underwent ERA-guided euploid embryo transfer (EET). Unexpectedly, it revealed that ERA could not optimize the reproductive outcomes in EET cycles, no matter in general infertile population or in patients with a history of previous failed embryo transfers.

## 1 Introduction

The embryo’s quality and endometrial receptivity are two vital factors for successful pregnancy. As the embryo’s quality could be identified by preimplantation genetic test (PGT) (1), endometrial receptivity is believed to be the last “barrier” (2). Endometrial receptivity refers to endometrial status that supports blastocyst acceptance. Endometrial receptivity array (ERA), a diagnostic molecular tool, could divide endometrium into “receptive” or “non-receptive” status by identifying the expression of 248 molecular genes (3). It was reported that 20% of infertile population and 25% of patients with recurrent implantation failure (RIF) suffered from a “non-receptive” endometrium (4). In addition, the accuracy of ERA was superior to endometrial histology and completely reproducible (5). Therefore, ERA was speculated to optimize the reproductive outcomes in patients who underwent *in vitro* fertilization (IVF) treatment, especially in patients with RIF, as it proposed a personalized optimal transfer time. However, no consensus has been reached yet. This review aims to investigate if ERA was effective in optimizing the reproductive outcomes in a systematic way. As controlling for the embryo’s quality would allow for a more accurate assessment, we only analyzed the effects of ERA in euploid embryo transfer (EET) cycles.

## 2 Methods

### 2.1 Study screening

**Figure 1:**
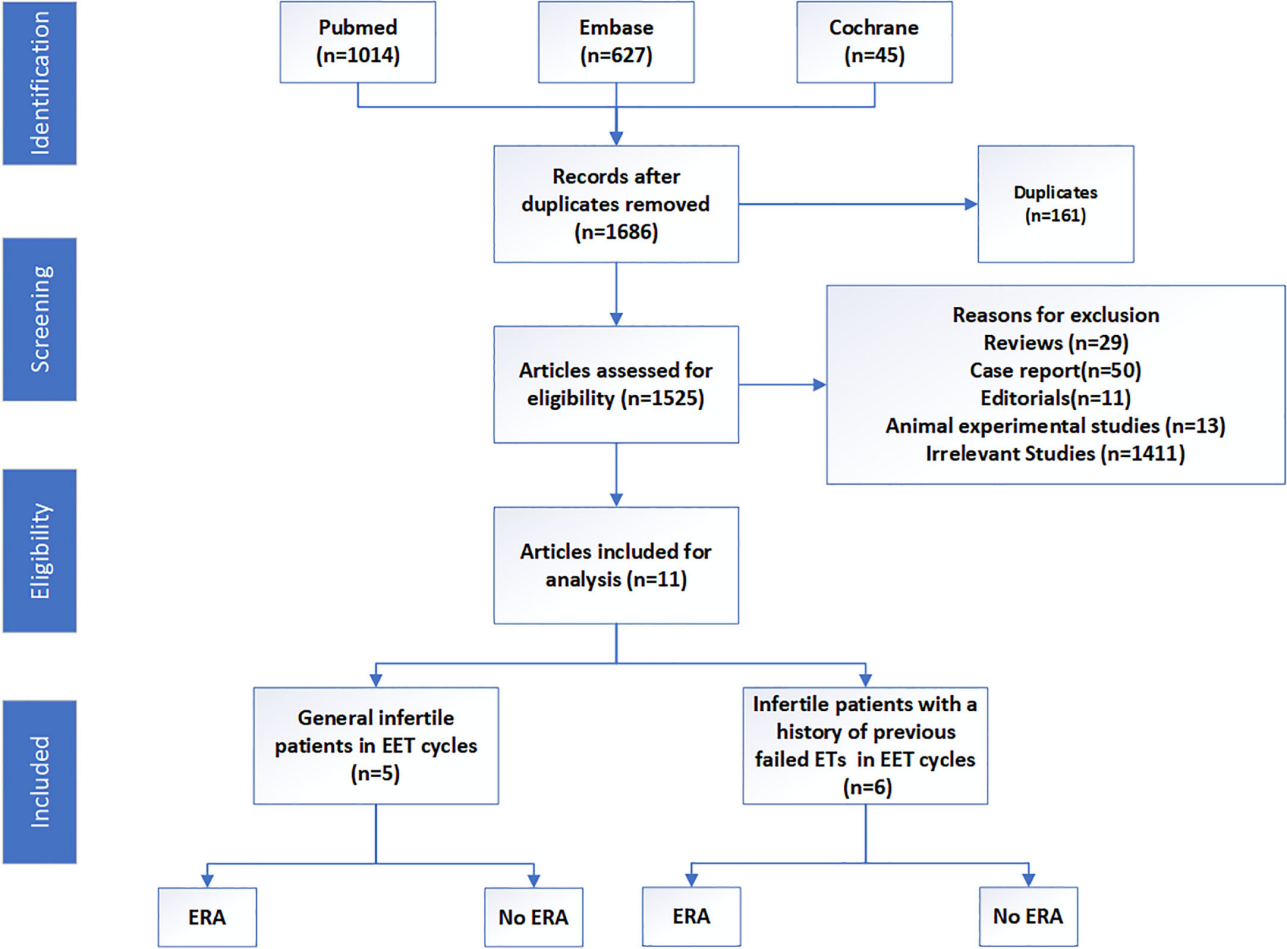
PRISMA flowchart.

We systematically searched PubMed, EMBASE, and Cochrane Central Register of Controlled Trials databases from inception to June 2023 according to our search strategy (**Supplementary Text 1**). The inclusion criteria were as follows: published in English language, irrespective of study design, and studies focusing on the effects of ERA in EET cycles. Review articles, case reports, editorials, animal experimental articles, and studies not related with the effects of ERA in EET cycles were excluded (**Figure 1**). 

### 2.2 The risk of bias assessment

Assessment of risk of bias was done by two independent researchers (MYW and WYC) according to the “modified Newcastle–Ottawa scoring items” (**Supplementary Table S1**). The scale mainly included five factors: sample representativeness, sampling technique, ascertainment of “receptive or non-receptive” diagnosis, quality of description of the population, and data completeness. Total scores ranged from 0 to 5, and studies were judged to be of low risk of bias (≥3 points) or high risk of bias (<3 points) (6).

### 2.3 Data extraction and analysis

The selected studies were comprehensively examined, and the relevant data were extracted according to our developed spreadsheet (MYW and WYC). The extracted data included author’s name, publication year, study year and country, study aim, study design, sample size, sample characteristics, embryo stage, number of transferred embryos, and outcome measures. The primary outcomes of interest were ongoing pregnancy rate (OPR) and live birth rate (LBR). Secondary outcomes of interest included implantation rate (IR), clinical pregnancy rate (CPR), biochemical pregnancy loss rate (BPLR), and miscarriage rate (MR). This review was conducted according to the Preferred Reporting Items for Systematic Reviews and Meta-Analyses guidelines (7).

## 3 Results

### 3.1 Studies selection and characteristics

In total, 1,686 articles were obtained, and 1,525 articles remained after 161 duplicates were removed. Of these, 29 reviews, 50 case reports, 11 editorials, and 13 animal experimental studies were excluded. Subsequently, 1,411 articles were found irrelevant and excluded. Finally, 11 articles were included in this review, which consisted of one double-blind randomized clinical trial, two prospective reports, and eight retrospective studies. These studies were all published between 2018 and 2023, while most studies were from the Occident. The population were mainly divided into two groups: general infertile patients and those with a history of previous failed embryo transfers. The great heterogeneity among these studies precluded the possibility of a meta-analysis. However, we also tried to present a comprehensive review of the implications of ERA in EET cycles. The basic clinical characteristics and reproductive outcomes of these studies are presented in **Table 1**.

**Table 1:** Reproductive outcomes in ERA group and non-ERA group.

Reference	Study year/country	Study design	Inclusion criteria	Cycle	Study group	No. of cases	Age	Embryo stage	No. of ET	OPR or LBR	IR	CPR	BPLR or MR
Leondires, 2018 (8)	2016–2017 Spain	Prospective	≥ One failed euploid ET	FET (HRT)	PGT, ERA	15	34.5 ± 4	^–^	^–^	73.30%	87.50%	^–^	BPLR6.7%, MR20%
Tan, 2018 (9)	2014–2017 Netherlands	Retrospective	RIF, One prior failure, No prior failures	FET (HRT)	PGT, ERA	17	36.8 ± 4.1	blastocyst	1.09 ± 0.3	64.70%	76.50%	^–^	^–^
					PGT, no ERA	26	37.6 ± 3.9		1	42.30%	53.8%	^–^	^–^
Rosen, 2019 (10)	– the USA	Retrospective	General infertile population	FET (HRT)	PGT, ERA (receptive)	200	^–^	blastocyst	^–^	55%	^–^	74%	MR 9%
					PGT, ERA (non-receptive)	147				52.40%	^–^	72.70%	MR 10.9%
Neves, 2019 (11)	2012–2018 Portugal	Retrospective	≥ 1 failed euploid–ET	FET (HRT)	PGT, ERA	24	39.25 ± 3.99	blastocyst	1.13 ± 0.34	^–^	55.60%	58.30%	^–^
					PGT, no ERA	119	39.18 ± 3.80		1.18 ± 0.38	^–^	65.00%	70.60%	^–^
Bergin, 2020 (12)	2014–2019 the USA	Retrospective	General infertile population	^–^	PGT, ERA after PSM	99	36.92 ± 3.64	blastocyst	1	51.52%	^–^	^–^	^–^
					PGT, no ERA after PSM	176	36.79 ± 3.81			56.82%	^–^	^–^	^–^
Cozzolino, 2020 (13)	2013–2018 Italy	Retrospective	RIF	Natural or HRT	M-RIF	2110		blastocyst	Total ET	^–^	^–^	^–^	^–^
					No PGT, no ERA	1840	37.9 (37.7–38.1)		2636	35.89%	34.20%	^–^	^–^
					PGT, no ERA	144	38.2 (38.0–38.5)		183	58,36.25%	38.20%	^–^	^–^
					No PGT, ERA	111	38.6 (38.3–38.9)		160	84,45.9%	40%	^–^	^–^
					ERA, PGT	15	38.5 (38.1–39.0)		21	7,33.33%	33.30%	^–^	^–^
					S-RIF	488		blastocyst		^–^	^–^	^–^	^–^
					No PGT, no ERA	408	38.5 (38.2–38.7)		591	201,34.01%	34.80%	^–^	^–^
					PGT, no ERA	53	38.3 (38.1–38.6)		72	14,40%	39.80%	^–^	^–^
					No PGT, ERA	23	38.9 (37.8–41.9)		35	26,36.11%	37%	^–^	^–^
					ERA, PGT	4	37.9 (37.7–38.5)		6	2,33.33%	33.30%	^–^	^–^
Rao, 2021 (14)	2014- 2019 India	Retrospective	RIF	^–^	PGT, ERA	79	^–^	blastocyst	1		53%	^–^	^–^
					PGT, no ERA	54			1	^–^	47%	^–^	^–^
					No PGT, no ERA	189			1	^–^	42%	^–^	^–^
Fodina, 2021 (15)	2017–2020 Latvia	Retrospective	RIF in ICSI cycles	^–^	No PGT, no ERA	72	34.0 (37.0–32.0	blastocyst	^–^	^–^	^–^	44.40%	BPLR 5.6%; MR1.4%
					No PGT, ERA	22	36.0 (38.0–34.0)			^–^	^–^	36.40%	BPLR 4.5%; MR9.1%
					PGT, no ERA	87	35.0 (37.0–33.0)			^–^	^–^	49.30%	BPLR 17.9%; MR4.5%
					PGT, ERA	72	34.0 (38.0–32.5)			^–^	^–^	55.60%	BPLR 1.4%; MR1.4%
Riestenberg, 2021 (16)	2018–2019 the USA	Prospective	General infertile population	FET (HRT)	PGT, ERA	147	36.9 ± 3.8	blastocyst	1	LBR 56.5%	^–^	67.40%	BPLR 15.4%; MR 15.2%
					PGT, no ERA	81	34.9 ± 3.8		1	LBR 55.6%	^–^	65.40%	BPLR 14.8%; MR 13.2%
Doyle, 2022 (17)	–the USA	Retrospective	General infertile population	FET	PGT, ERA	307	36.7 ± 4.1	blastocyst	1	44.60%		54.10%	
					ERA receptive N (%)	125				LBR48.8%		59.20%	
					ERA non-receptive N (%)	182				LBR41.7%		51.60%	
					PGT, no ERA	2284	36.7 ± 4.3		1	51.30%		58.90%	
Doyle, 2022 (18)	– the USA	Double-blind, randomized, multicenter clinical trial	General infertile population	FET	PGT, ERA	381	34.7 ± 2.7	blastocyst	1	LBR 58.5%	77.20%	68.80%	
					PGT, no ERA	386	34.5 ± 2.7		1	LBR 61.9%	79.50%	72.80%	

The superscript “–” refers to missing data.

ERA: 1663.

### 3.2 ERA could not optimize the reproductive outcomes in EET cycles in the general infertile population

In 2019, Rosen (10) conducted a retrospective study to describe the reproductive outcomes of 347 patients who had ERA-guided EET. The patients with non-receptive endometrium (42.3%) had a modified protocol accordingly. As a result, the patients with receptive endometrium and those with non-receptive endometrium had similar ongoing pregnancy rate (55% vs. 52.4%) and miscarriage rate (9% vs. 10.9%). However, this article did not include a control group who did not undergo ERA. Therefore, the effects of ERA may not be well demonstrated.

In 2021, Bergin (12) conducted a retrospective study, which included 110 patients who underwent ERA in EET cycles and 2,550 controls (non-ERA in EET cycles). Following propensity score matching (PSM), 99 patients in the study group were successfully matched to 176 controls. The results revealed that the live birth rate did not differ in the ERA group (n=99) and the non-ERA group (n=176) (51.52% vs. 56.82%). In the same year, Riestenberg conducted a prospective study (16), which included patients who had their first EET cycles with or without ERA. The results showed that the live birth rate was not significantly different between 147 patients with ERA and 81 controls without ERA (56.6% vs. 56.5%). In 2022, Doyle (17) conducted a large retrospective cohort study, which enrolled patients who underwent EET cycles guided by ERA (n=307) or not (n=2,284). The non-receptive rate was 59.1% in the ERA group. However, there was no difference in the live birth rate between ERA group and non-ERA group (44.6% vs. 51.3%). Doyle (18) also conducted a double-blind, multicenter, randomized clinical trial to compare the live birth rate in patients who had ERA (n = 381) or not (n = 386) in their single EET cycle. As a result, there were no significant differences in the live birth rate (58.5% vs. 61.9%), biochemical pregnancy rate (77.2% vs. 79.5%), and clinical pregnancy rate (68.8% vs. 72.8%) in both groups.

### 3.3 ERA could not optimize the reproductive outcomes in EET cycles in patients with a history of previous failed embryo transfers

At the beginning, some studies reported a favorable trend for the reproductive outcomes in ERA-guided EET cycles in patients with a history of previous failed embryo transfers. In 2018, Leondires (8) conducted a prospective pilot study, which included patients who underwent ERA-guided EET with a history of previous failed EET (n=15). It revealed that the non-receptive rate was up to 86.7%, and the ongoing pregnancy rate could reach 73.3% with a personized embryo transfer (pET) guided by ERA. However, this article did not include a control group without ERA, and the sample size was too small. In the same year, Tan (9) conducted a retrospective study, which included patients with RIF (n=30), or one prior failure (n=13) who underwent EET or not. The results revealed that the patients whose embryo transfer guided by ERA (n=17) had increased implantation (76.5 vs. 53.8%) and ongoing pregnancy rates (64.7 vs. 42.3%) compared with those not (n=26). However, the differences were not statistically significant.

In 2019, Neves (11) conducted a retrospective study to compare the reproductive outcomes of patients with previous failed embryo transfer (≥ 1 previous failed EET) who underwent ERA (n=24) or not (n=119). The results revealed that the implantation rate (55.6% vs. 65.0%) and the pregnancy rate (58.3% vs.70.6%) did not show significant differences in both groups. In 2020, Cozzolino (13) conducted a retrospective multicenter cohort study in which patients classified as moderate RIF (n=2110) and severe RIF (n=488) were enrolled. Moderate RIF was defined as implantation failure after receiving at least three embryos transferred in different single embryo transfers without PGT or ERA, while severe RIF consisted of patients who failed after receiving at least five embryos transferred. The included patients in the moderate/severe RIF group were divided into four groups: group I, no PGT, no ERA (n=946/n=201); group II, PGT, no ERA (n=58/n=14); group III, no PGT, ERA (n=84/n=26); and group IV, ERA, PGT (n=7/n=2). The authors concluded that the reproductive outcomes did not differ between those who underwent ERA and those who did not. In 2021, Rao (14) retrospectively reviewed the reproductive outcomes of patients with RIF (n=322), who were divided into three groups: group I, ERA, PGT (n=79); group II, PGT, no ERA (n=54); and group III, no PGT, no ERA (n=189). Similarly, there was no difference in the implantation rate among the three groups (53% vs. 47% vs. 42%). In the same year, Fodina (15) conducted a retrospective study, which included 253 cycles with a history of RIF. The patients were divided into four groups: group I, no PGT, no ERA (n = 72); group II, PGT, no ERA (n = 87); group III, PGT, ERA (n = 72); and group IV, no PGT, ERA (n = 22). The results also showed that ERA failed to optimize the reproductive outcomes.

## 4 Discussion

Apart from the embryo’s quality, the endometrial receptivity is extremely important, as it is the “soil” for the “seed” (the embryo). To the best of our knowledge, this review may first analyze the effects of ERA, eliminating embryo quality as a confounder. The present review was based on 11 available studies including 7,581 patients of whom 1,663 were evaluated by ERA. It revealed that ERA could not optimize the reproductive outcomes in both general infertile patients and patients with a history of previous failed embryo transfers in EET cycles.

The conclusion of the present review was consistent with previous studies, which stated that ERA was not effective in non-EET cycles. Recently, a retrospective multicenter cohort study with large sample size demonstrated that the LBR and cumulative LBR were even higher in non-ERA group than ERA group in 3,239 autologous transfers, even when considering possible confounders (19). A re-analysis of data from randomized controlled trial also revealed that ERA-guided pET actually reduced rather than increased the LBR in non-EET cycles (20). Another meta-analysis also stated that the LBR and OPR were comparable between the ERA and the non-ERA groups in infertile patients, even in the subgroup of patients with previous embryo transfer failures (21). The underlying mechanisms of ERA’s ineffectiveness may be as follows. First, whether the endometrial status was receptive in the post-ERA cycle was unknown, as endometrial biopsy of a modified cycle was not performed. Furthermore, not all non-receptive endometrium is pathological; an ERA-guided protocol may be not beneficial for successful pregnancy (22). Second, implantation is a multifactorial and complex process; other factors may also affect the endometrial status. One previous study found that only when ERA is used in conjunction with immune profiling that reproductive outcomes can be predicted (11). This indicated that ERA-guided protocol solely is not sufficient for successful implantation.

It should be noted that there remains conflicting data as to the impacts of ERA, especially in patients with RIF. Simon stated that ERA-guided pET group had a higher pregnancy rate per ET and a trend to a higher implantation rate and ongoing pregnancy rate (23). Luo’s (24) and Liu’s (6) meta-analysis both stated that ERA was not beneficial in patients without RIF or good-prognosis patients. However, it may improve the reproductive outcomes of patients with RIF. The underlying mechanism for the conflicting results among different literature may be as follows. First, the patients’ characteristics such as age and body mass index (BMI), IVF cycles protocol, embryo transfer protocol, number of transferred embryos, and the interval of ERA biopsy to pET varied among articles, potentially generating bias in the estimation of the impacts of ERA (25–27). Second, genetic abnormalities, tubal factors, immunological factors, and thrombophilias besides endometrial pathologies were all underlying causes of RIF (28). These would also become confounding factors in evaluating the effects of ERA in RIF. Last but not least, RIF has no clear definition (29). It was reported that when RIF was defined as two or more implantation failures, the live birth rate was significantly lower than when RIF was defined as three or more implantation failures (30). This may be also the reason of the conflicting data about ERA’s impacts.

A significant limitation of the present review is that most of the included studies were retrospective, and only one RCT was available. However, we conducted separate subgroup analyses according to the design of the studies. Another limitation was the heterogeneity of the studies included. Therefore, the results of the present review should be interpreted cautiously, and more randomized controlled trials were required to explore the potential effects of ERA.

## 5 Conclusion

ERA-guided embryo transfers have no beneficial effects in optimizing the reproductive outcomes in general infertile population and patients with a history of previous failed embryo transfers in EET cycles.

## Author contributions

YM and YW drafted the manuscript and participated in data collection and analysis. XK and XL performed the statistical analysis. YL and FW participated in its design and coordination. All authors contributed to the article and approved the submitted version.
