# Supplementary material for: Does endometrial receptivity array improve reproductive outcomes in euploid embryo transfer cycles? a systematic review
Source: Front Endocrinol (Lausanne). 2023 Oct 23;14:1251699. doi: 10.3389/fendo.2023.1251699 (PMC10641275; doi:10.3389/fendo.2023.1251699)
Supplement: Supplementary file 1 [file Table_1.docx]

Sup Table 1 The risk of bias assessment

| Authors and year Sample | representativeness | Sampling technique | Ascertainment of non-receptive Diagnosis | Quality of Description of the Population | Incomplete Outcome data | Total score | Risk of bias |
| --- | --- | --- | --- | --- | --- | --- | --- |
| Leondires,2018 | 0 | 1 | 1 | 1 | 1 | 4 | low |
| Tan,2018 | 0 | 0 | 1 | 1 | 1 | 3 | low |
| Rosen,2019 | 1 | 0 | 1 | 1 | 1 | 4 | low |
| Neves,2019 | 1 | 0 | 1 | 1 | 1 | 4 | low |
| Bergin,2020 | 1 | 0 | 1 | 1 | 0 | 3 | low |
| Cozzolino,2020 | 1 | 0 | 1 | 1 | 0 | 3 | low |
| Rao,2021 | 1 | 0 | 1 | 1 | 0 | 3 | low |
| Fodina,2021 | 1 | 0 | 1 | 1 | 1 | 4 | low |
| Riestenberg, 2021 | 1 | 1 | 1 | 1 | 1 | 5 | low |
| Nicole,2022 | 1 | 0 | 1 | 1 | 0 | 3 | low |
| Nicole,2022 | 1 | 1 | 1 | 1 | 0 | 4 | low |

The superscript “-” refers to missing data.
